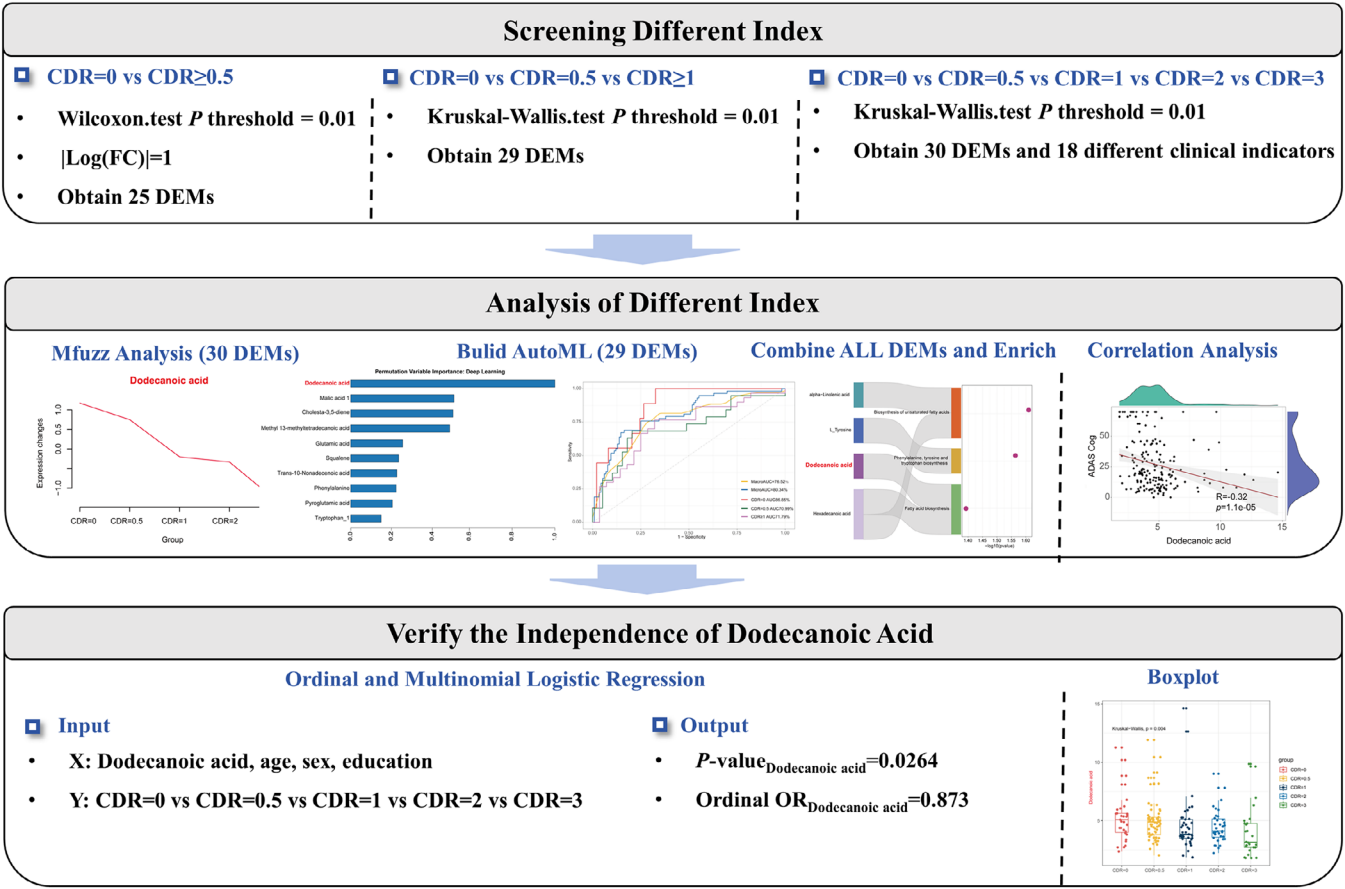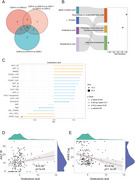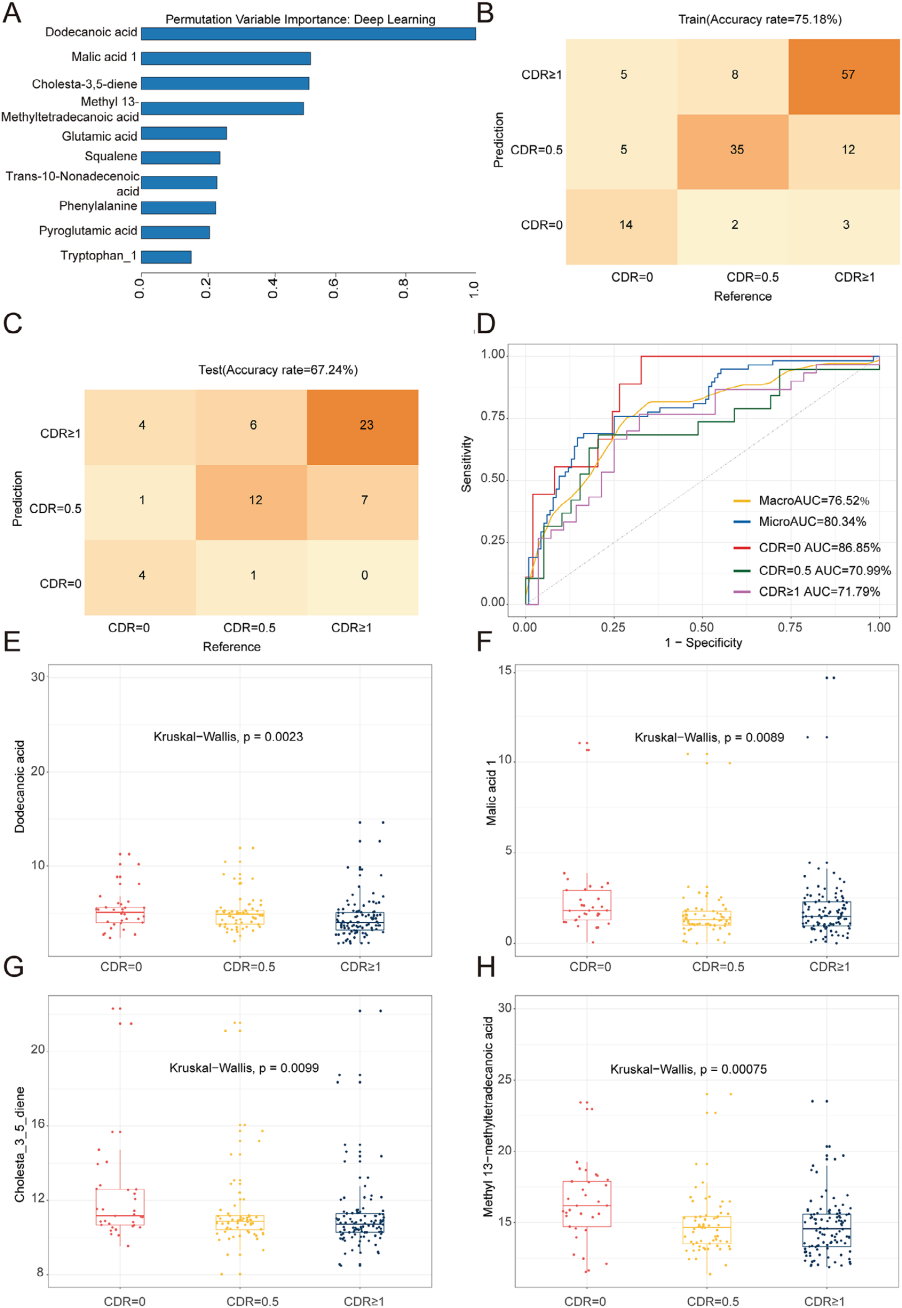# Fingernail‐based Metabolomics Reveals a Stepwise Decline in Dodecanoic Acid Associated with Alzheimer's Disease Progression

**DOI:** 10.1002/alz70856_101814

**Published:** 2025-12-25

**Authors:** Wenbo Zhang, Jiani Wu, Zhangjing Deng, Weihua Yu, Yang Lü

**Affiliations:** ^1^ The First Affiliated Hospital of Chongqing Medical University, Chongqing, Chongqing, China; ^2^ Chongqing Medical University, Chongqing, Chongqing, China

## Abstract

**Background:**

Fingernail metabolomics provides a novel, non‐invasive platform that captures long‐term biochemical fluctuations for identifying reliable biomarkers for dementia and mild cognitive impairment (MCI) due to Alzheimer's disease (AD).

**Method:**

This study enrolled 199 participants stratified by Clinical Dementia Rating (CDR) scores (0, 0.5, 1, 2, and 3). Fingernail clippings were collected and subjected to gas chromatography–mass spectrometry–based metabolomic analysis. Differentially expressed metabolites (DEMs) were identified across cognitive groups using clustering, ordinal logistic regression, and machine learning approaches. Pathway enrichment and correlation analyses were performed to explore underlying disease mechanisms and clinical relevance.

**Result:**

Thirty DEMs were discovered across the five CDR categories. Notably, dodecanoic acid demonstrated a pronounced decline from cognitively normal individuals (CDR = 0) to advanced AD (CDR = 3). After adjustment for age, sex, and education, dodecanoic acid remained independently associated with disease severity (*p* = 0.0264, *OR* = 0.873). Importantly, within each CDR category (0.5, 1, 2, and 3), dodecanoic acid levels showed no significant differences between individuals with and without 18F‐AV45 PET‐confirmed amyloid pathology (all *p* > 0.05). Correlation analysis indicated that lower levels of dodecanoic acid were linked to greater cognitive impairment (AVLT‐IR: *r* = 0.29; ADAS‐Cog: *r* = –0.32). Pathway enrichment highlighted significant disruptions in fatty acid metabolism, suggesting an energy regulation deficit in AD. A deep learning model trained on 29 DEMs achieved a micro‐AUC of 80.34%, validating dodecanoic acid and related metabolites (e.g., cholesta‐3,5‐diene) as strong diagnostic indicators.

**Conclusion:**

Dodecanoic acid emerges as a critical biomarker reflecting disrupted fatty acid metabolism in AD progression. By leveraging fingernail metabolomics for long‐term metabolic profiling, this non‐invasive strategy offers a scalable approach for early diagnosis, staging, and monitoring of neurodegenerative diseases.